# The effects of integrated traditional Chinese and western medicine rehabilitation programs on post-acute ankle sprain: A randomized controlled trial study protocol

**DOI:** 10.1371/journal.pone.0318535

**Published:** 2025-01-30

**Authors:** Hang Gao, Xiao Chen, Jiayi Ren, Xinglai Zhang, Yuqian Hu, Zhao Ma, Yuanjia Gu, Jiming Tao, Weian Yuan

**Affiliations:** 1 Rehabilitation of Shuguang Hospital Affiliated to Shanghai University of Traditional Chinese Medicine, Pu Dong New District, Shanghai, China; 2 Shi’s Traumatology Medical Center of Shuguang Hospital Affiliated to Shanghai University of Traditional Chinese Medicine, Pu Dong New District, Shanghai, China; University of Tehran, ISLAMIC REPUBLIC OF IRAN

## Abstract

**Background:**

Ankle sprain is a common clinical disease, which has the highest incidence rate among joint and ligament injuries. And acute ankle sprains can easily develop into chronic ankle instability, thereby increasing the difficulty of treatment. The current clinical guidelines for post-acute ankle sprains are still controversial. Pain and functional impairment are the most significant symptoms after ankle sprain. The main purpose of this study is to explore the safety and effectiveness of the intervention of integrated traditional Chinese and Western medicine rehabilitation programs on post-acute ankle sprains.

**Methods:**

This study is a single center, prospective, intervention randomized controlled trial with a control group. 174 patients of 18–35 years old with post-acute ankle sprain will be included. A randomized controlled study is conducted and divided into a control group and an experimental group. The control group receive routine treatment, while the experimental group receive integrated traditional Chinese and western medicine rehabilitation programs. The intervention lasted for a total of 2 weeks. The main outcome measures are Visual Analog Scale and Short Form McGill Pain Questionnaire; the secondary outcome measures are the Foot Ankle Ability Assessment Scale, AOFAS Ankle Posterior Foot Scale, torque and gait. This study protocol aims to evaluate the safety and effectiveness of integrated traditional Chinese and western medicine rehabilitation programs by comparing the results of two groups before and after treatment. This protocol will follow the SPIRIT guidance.

**Discussion:**

At present, there is a lack of rehabilitation management for post-acute ankle sprains. Therefore, this study has the potential to improve the healthcare for post-acute ankle sprains patients and might be used for future standardized evidence-based rehabilitation concepts.

**Trial registration:**

The trial was registered at Chinese Clinical Trial Registry https://www.chictr.org.cn/showproj.html?proj=234465 (Registration No.: ChiCTR2400087456). Date: 2024-07-29.

## Introduction

Ankle sprain is one of the most common injuries during our clinic career. Ankle sprain often occurs with foot inversion or adduction, and most often happens at the lateral ankle [1]. The risk factors for ankle sprains include limited range of motion of the ankle joint, poor coordination function, weakened balance function [2,3], decreased proprioception and insufficient muscle strength [4–6], and even decreased cardiovascular and pulmonary function [4,7]. Exercise is the highest risk factor for ankle sprain occurrence [8]. About 40% of patients develop into chronic ankle sprains with persistent symptoms [9,10]. Usually, we consider the acute period refers to the first 1–2 weeks after injury. And, the term “post-acute” replace "subacute" to characterize the time after the acute period to the 12-month point. Literature has found that 60% of individuals achieve resolution of activity limitations and participation restrictions by the 12-month point [11]. And often we tend to refer to chronic ankle sprain as ankle instability which prolongs patient pain, affects patient’s life, enlarges treatment costs and social burden [12]. Thereby, we consider the post-acute period as an important phase for ankle sprain recovery.

Due to the fact that the ankle joint is an important load-bearing bone joint, its stability and function are crucial for daily activities and exercise. Although there are currently multiple treatment methods for ankle sprains, there are differences in the efficacy and safety of different methods [7,13]. The study of effective treatment methods for post-acute ankle sprains has important clinical significance.

When ankle sprain occurs, the “RICE” principle (rest, ice application, compression and elevation) can be used for treatment. The initial focus of the rice principle was on reducing pain while restoring strength and range of motion. However, the evidence supporting this method is limited [7,14]. Physical therapy is also believed to promote the recovery process, but the effect is slow and time-consuming [15]. Evidence from RCTs of moderate quality indicate that daily physical therapy is better tolerated than longer, less frequent physical therapy sessions [16]. Extracorporeal shock wave is considered one of the treatment methods for soft tissue injuries in the feet and ankles [17,18]. The sustainability of ESWT treatment efficacy may depend on energy levels and the severity of ankle sprains, which should be studied in future research [19]. From the perspective of traditional Chinese medicine treatment, acupuncture is considered a complementary and alternative therapy to modern medicine [20,21] and Previous systematic reviews have shown that acupuncture treatment for ankle sprains has a certain therapeutic effect [21–23]. However, the evidence for acupuncture treatment of ankle sprains is still uncertain due to significant heterogeneity [22,24]. Manual training can help recover from ankle sprains, which is also recommended in the guidelines [25]. Manual training can alleviate pain and improve gait [26]. The involvement of neuromuscular training programs in manual training can effectively prevent the recurrence of ankle sprains [27]. considering that neuromuscular and proprioceptive training is safe and effective, the guidelines also recommend implementing neuromuscular training as soon as possible after ankle sprains [28–30]. Surgery is one of the strategies for treating ankle sprains. Kerkhoffs et al. found similar functional and subjective symptom outcomes when comparing surgical intervention and conservative treatment for lateral ankle ligament complex injuries [31]. Severe tibiofibular syndesmosis injury or combined with triangular ligament rupture can benefit from surgical fixation. Ankle joint fracture combined with lower tibiofibular joint injury requires surgical fixation of the fracture. However, repairing ligament injuries simultaneously is controversial [32].

In a word, there are many methods to deal with an ankle sprain. Traditional treatment methods such as “RICE” principle will be first adopted which can alleviate pain and swelling to a certain extent, but more effective treatment methods are needed for serious situations such as ligament damage. However, the latest evidence-based clinical guidelines do not recommend the best treatment for ankle sprains [7]. Based on the above reasons, we draft a protocol on the integrated traditional Chinese and western medicine rehabilitation programs (TCAWM) for the treatment of post-acute ankle sprains. Our clinical research aims to evaluate whether TCAWM can be more effective treatment methods for post-acute ankle sprains, in order to improve the rehabilitation effect and quality of life of patients.

## Methods

### Design

**Fig 1:**
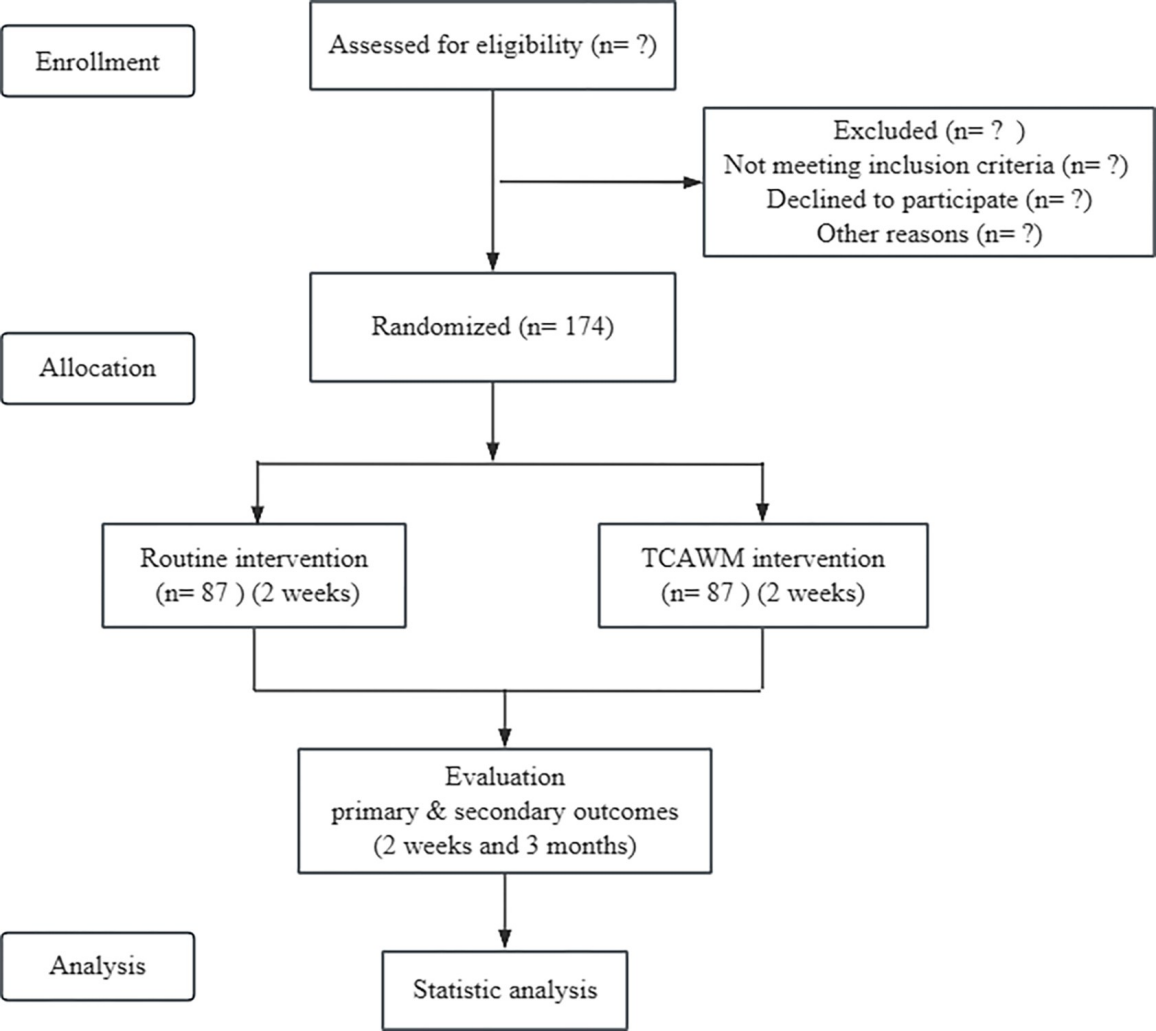
Study flow chart.

The study is a randomized controlled trial (RCT). A total of 174 post-acute ankle sprain patients will be recruited and randomly divided into the intervention group and the control group at a ratio of 1:1. Recruitment of eligible patients will begin from January 2025. Participants of the intervention group will be treated with TCAWM and participants of the control group will be treated with routine treatment. The whole study period will be 2-week including baseline and 2-week treatment. The study design is illustrated in the flow chart in Fig 1. The study protocol was approved by the Regional Ethics Review Committee of Shuguang Hospital (number: 2024-1535-118-01).

### Participant

Patients will be recruited in Shuguang Hospital, through bulletin board posts and surrounding communities through advertising (posting notices and online advertisement). All eligible participants will be tested in Shuguang Hospital and operated by doctor responsible for recruitment. Ensuring consistency and replicability in our study, this protocol will include structured assessment tools that will be administered by trained research staff. All personnel involved in the screening process will undergo comprehensive training to ensure they understand the inclusion and exclusion criteria thoroughly. This training will cover how to administer the screening tools, interpret responses, and make decisions based on the criteria. Meanwhile, we will maintain detailed records of the screening process, including the number of individuals screened, the reasons for exclusion, and any challenges encountered. This documentation will be included in our final report to provide transparency and facilitate replicability. The eligible participants must meet the following criteria.

#### Diagnostic criteria

The participant needs to meet the diagnostic criteria. Referring to the "Diagnostic and Therapeutic Efficacy Standards for Traditional Chinese Medicine Diseases" issued by the National Administration of Traditional Chinese Medicine and the Clinical Practice Guidelines for the International Classification of Function, Disability, and Health of the American Physical Therapy Association Orthopedic Branch for Ankle Ligament Sprains (2021 Edition), it is proposed that: ① There is a clear history of ankle trauma; ② After spraining, there is obvious tenderness or subcutaneous bruising in the ankle joint, accompanied by swelling and pain, limited weight-bearing, and limping; ③ Limited joint mobility; ④ Ottawa principle is negative, X-ray examination shows no fracture or dislocation.

### Inclusion criteria

① Those who meet the diagnostic criteria for ankle sprains, in the post-acute period, and have an injury grade of I or II [33];

② Age range from 18 to 35 years old, regardless of gender [34–37];

③ Cumberland Ankle Instability Scale score ≤ 24 points;

④ Those who have not received any other treatment before treatment and are willing to receive conservative treatment shall sign an informed consent form.

### Exclusion criteria

① Both ankles sprained;

② Individuals with skin damage or skin disease in the affected area;

③ Ankle fracture, dislocation, severe osteoporosis and diseases that may affect muscle strength, such as diabetes and rheumatoid arthritis;

④ Subjectively unwilling to accept the experimenter by the patient;

⑤ Use antipyretic, analgesic, sedative, or steroid drugs in the past week;

⑥ Have a history of cardiovascular and cerebrovascular diseases, central nervous system tumor diseases, mental illness, or head injury;

⑦ Individuals with contraindications for physical therapy such as metal objects in the ankle.

### Intervention

A randomized controlled study is conducted and divided into a control group and an experimental group. The control group receive routine treatment, while the experimental group receive the integrated traditional Chinese and western medicine rehabilitation programs (TCAWM), once a day and five times a week, for a total of two weeks of intervention. The intervention schedules for each group are shown in Table 1 Intervention Schedules.

**Table 1:** Intervention schedules.

Period	Screening	Allocation	Intervention		Follow up
Week	-2week	-1week	0week	2week	3 months
Eligibility screening	✓				
Informed consented	✓				
Demographics	✓				
Diagnosis	✓				
Medical history	✓				
Randomization and allocation		✓			
VAS			✓	✓	✓
SF-MPQ			✓	✓	✓
FAAM			✓	✓	✓
AOFAS Ankle Posterior Foot Scale			✓	✓	✓
Torque			✓	✓	✓
Gait			✓	✓	✓
Adverse events				✓	✓

VAS, Visual Analog Scale; SF-MPQ, Short form McGill Pain Questionnaire; FAAM, Foot and Ankle Ability Assessment Scale; AOFAS Ankle Posterior Foot Scale, American Orthopedic Foot and Ankle Association Ankle Posterior Foot Scale.

#### The control group

Routine intervention: including pressure cold compress, shock wave, microwave and other physical therapies; Soft tissue stretching and relaxation, passive and active joint activity of the ankle, and strength training of muscles around the ankle.

#### The experimental group

TCAWM intervention:

① Physical therapy: infrared dose of 300w, distance of 20cm, irradiate the sick ankle; The shockwave, energy flow density is 0.15mJ/cm^2^, with a pulse count of 2000.

② Exercise training:

a. Muscle strength training: Use elastic bands for ankle dorsiflexion, plantar flexion, inversion, and eversion resistance training, maintain for 10 seconds, perform 20 times in all directions, 2 groups per day;

b. Joint range of motion training: Participants engage in active ankle dorsiflexion, plantar flexion, inversion, and eversion, maintaining a maximum range of motion for 10 seconds, and performing 20 movements in each direction; and perform clockwise and counterclockwise rotation movements of the ankle joint, with gentle, slow, and uniform speed, each rotating 20 times. Two groups per day;

c. Balance training board training: Stand on the balance board, maintain balance with both feet without additional support for 30 seconds, train 20 times, and then maintain balance with one foot without additional support for 20 seconds, alternating between the two feet, each training 20 times, with 2 groups per day;

d. Stable reinforcement training: Conduct in place takeoff training, turn back runs (distance of 100m), each group of takeoff training 50 times, turn back runs 10 times (once in a row). Two groups per day.

③ Acupuncture: local acupoints are the main acupoints, and the acupoints selected are GB40, GB41, BL62, SP5, KI6 and KI5. Warm acupuncture and moxibustion and electroacupuncture can be used.

④ Massage: Select acupoints around the ankle joint, such as GB34, GB40, GB39, KI6, and BL62. The techniques used include pressing, kneading, one finger Zen pushing, stretching, shaking, rubbing, etc. The patient is placed in a supine position, with the physician standing on the affected side and using the thumb massage method to apply pressure to the ankle. First, from the affected area to the surrounding area, and then from the outer ankle through the outer side of the calf to GB34 acupoint, massage three times, with a focus on GB34, GB40, GB39, KI6, BL62 and other acupoints, with a degree of soreness and swelling. Finally, pull and extend the ankle joint several times and perform small internal and external rotations; Then perform ankle joint rocking several times; Wipe the back of the foot using the small thenar wiping method, and extend from the ankle to the calf.

### Outcomes measures

#### Primary outcomes

① Visual Analog Scale (VAS) [38,39]. VAS is used to evaluate the degree of pain. Draw a 10cm horizontal line on the paper with a starting point of 0 and a ending point of 10, marking 0 as painless and 10 as unbearable pain. Mark the corresponding scale every 1cm in the middle of the line. Ask the subjects to mark any position on the horizontal line based on their pain perception, as the degree of VAS pain in the subjects.

② Short form McGill Pain Questionnaire (SF-MPQ) [38,39]. SF-MPQ is used to evaluate pain sensation, with a total of 47 items. The first 11 items evaluate the degree of pain sensation (PRIA), and the 12–15 items evaluate the emotional state of pain (PRIB). The pain level of each item is described as painless (0 points), mild (1 point), moderate (2 points), and severe (3 points). In addition, both pain status and visual analogue scores are now included in the evaluation of overall pain status.

#### Secondary indicators

① Foot and Ankle Ability Assessment Scale (FAAM) [39,40]. FAAM is a method for assessing the degree of daily living ability limitation and ankle mobility disorders, consisting of 21 scores from daily living activities and 8 scores from the Independent Movement Disorder Scale.

② AOFAS Ankle Posterior Foot Scale [41]. Using the American Orthopedic Foot and Ankle Association (AOFAS) Ankle Posterior Foot Scale as a reliable and effective tool for detecting ankle ligament injuries. This standard consists of two main parts, mainly including pain, function and autonomous activity, support, flexion/extension, inward/outward rotation, ankle and foot stability, etc., with a maximum score of 100 points. The two major scores are each 50 points, with a maximum score of 100 points, excellent (90–100 points), good (75–89 points), average (50–74 points), and poor (<50 points). The larger the score, the better the ankle joint function and symptoms.

③ Torque [42]: Peak torque (PT); Peak torque to body weight ratio (PT/BW); Average power (AP); The ratio of bending to elongation peak torque (F/E); The ratio of homonymous contralateral muscles. The testing instrument uses the BIODEX multi joint constant velocity force testing and training system from the United States, and the raw data obtained from the test is automatically generated by the computer. The subject is in a supine position on the seat, and according to their height, body shape, etc., the equipment is adjusted and fixed strictly in accordance with the equipment safety manual. During testing, adjust the seat height and power head scale according to the parameters provided by the software, align the foot movement plane with the foot pedal movement plane, keep the outer ankle in a straight line with the power head rotation center, use nylon ropes to fix the subject’s thighs and feet to the accessories, and perform gravity compensation before testing. Firstly, under the condition of an angular velocity of 60°/s, the subjects were subjected to three maximum contraction exercises of ankle dorsiflexion and plantarflexion. After familiarizing themselves, the subjects rested for 30 minutes to sequentially perform isokinetic muscle strength tests on the healthy and affected ankle joints. Under the conditions of an angular velocity of 30°/s, 60°/s, and 120°/s, active centripetal movements of ankle dorsiflexion and plantarflexion, ankle inversion, and eversion were performed, with 10 consecutive maximum contractions completed each time.

④Gait [43,44]: Step length; Step speed; Step frequency; Supporting phase; Plantar pressure; Ankle flexion and inversion angle [39]. Apply Maiwo Odonate 3D motion capture and gait analysis equipment to test the patient’s walking ability and plantar pressure. The collection end selects gait collection combined with plantar pressure detection mode. Based on the work framework and patch model recommended by the International Society of Biomechanics. The subjects are required to wear tight fitting clothing. Infrared reflector positioning (23 in total). The entire process is carried out by the same senior rehabilitation therapist. Before collection, indicate the patient’s walking area, which is 1.6 to 4.5 meters away from the camera arm. Place the camera arm horizontally and ensure that it is parallel to the ground. Firstly, instruct the patient to stand still at a position of about 2 meters and click on "Static Collection"; Then instruct the patient to stand at a position of about 4.5 meters, click the "Dynamic Collection" button, and instruct the patient to start walking normally on the trail after waving their arms in place.

### Participant safety

When patients participate in this study, they will be required to know the scheme of the whole study and sign the informed consent. To prevent and better treat any injury that may be caused by this study, the researcher will detect any potential adverse events (AEs) and truthfully record on the CRF form. In this study, AEs are mainly including dizziness, shortness of breath, palpitations. If an adverse event occurs, it will be dealt with according to the emergency plan. Researchers will initially determine the severity of AEs. Minor AEs will be treated by the attending physician. Serious AEs will be reported by the researcher to the ethics committee. The correlation between the event and intervention and severity will be evaluated.

### Quality control

The head of the research center will be responsible for the design, coordination and quality control in whole study. All researchers will receive uniform training before the period of data collection. Throughout the research period, a data monitoring of committee independent of researchers and sponsors will extract 10% of case reports from and check the data every three months. The committee will check logic problems, determination of test values, abnormal safety indicators after treatment, vacancy values, compliance, standardization, integrity, consistency, etc. The committee will consist of clinicians, statistician, microbiologist, psychologist, and ethicist.

### Follow up

There will be a follow-up plan to evaluate the long-term efficacy and safety of TCAWM and routine treatment when the protocol ends, the research will follow up the patients after 3 months of the treatment. Participants will not receive any intervene during follow-up. At the end of follow-up, the investigator will make a contract with patients by telephone, E-mail and etc. They will be asked to come to reception room and have a test about VAS、SF-MPQ、FAAM、AOFAS、Torque and gait.

### Sample size

Using data from a previous study [45]. Comparison of sample size estimation formula for two sample means based on bibliometric data, with reference to relevant literature [a] for experimental group VAS (Visual Analog Scale) means μ_2_ = 0.04 after two weeks interventions, while control group VAS (Visual Analog Scale) means μ_1_ = 0.25, the standard deviation σ = 0.45. According to the formula, α = 0.05, β = 0.2, the sample size of each group is calculated to be n = 72, consider a 20% dropout rate, and the final sample size is 87 cases per group.


n1=n2=2×(Zα2+Zβ)2×σ2(μ2−μ1)2

### Randomization and allocation concealment

The patients who meet the diagnosis of post-acute ankle sprain and sign informed consent will be randomly assigned to control group and intervention group at a ratio of 1:1. Randomization will be computer generated by independent and uninformed statistical experts. Managers who do not participate in the recruitment and treatment place each number in an opaque envelope and keep it. After screening the patients, the doctor responsible for recruitment will contact the administrator who will send the file to the eligible participants and inform them of the allocation.

### Blinding

Due to the nature of the intervention, therapists and participants cannot be blinded. So we provide standard training to patients before they receive intervention, including informing them of the intervention methods and specific implementation, as well as self-assessment methods (VAS、SF-MPQ) for the efficacy after intervention; We have not only established self-assessment during the evaluation process, but also established third-party objective evaluations, and the third-party evaluators will also receive standard training including standardized use of equipment for evaluation to reduce the impact of intervention expectations. During the study, statisticians, administrator, data collectors and result evaluator will be blinded. After the statistics, the blinding method will be broken.

### Data collection and management

Two data administrators who do not belong to the research team and blinded to group allocation will be responsible for data entry and database establishment. We will implement a systematic approach to monitor participant retention throughout the study. This will include regular assessments of participant engagement and reasons for dropout, which will be documented and reported in our final analysis.Meanwhile,We will transparently report the extent and nature of missing data in our study. This will include the percentage of missing data for each variable, the reasons for missingness, and the methods used for imputation. This transparency will allow readers to assess the potential impact of missing data on our conclusions.To minimize dropouts, we will implement strategies aimed at enhancing participant engagement, such as regular follow-ups, reminders, and providing incentives for continued participation. All original data related to the study will be stored at Shuguang Hospital Affiliated to Shanghai University of Traditional Chinese Medicine and uploaded in real time to the China Clinical Trial Registry.

### Statistical analysis

For all outcomes, we will perform intent to treat analysis for our primary assessment of efficacy and a supplementary per protocol analysis. Use SPSS 21.0 statistical software for statistical analysis, including descriptive statistics such as mean, standard deviation. Quantitative data are analyzed using methods such as analysis of variance. Count data is analyzed using chi square test, Fisher’s exact test; Non parametric rank sum test and CMH chi square test is used for rank data. Set α = 0.05 as the significance test level, and when P<0.05, it indicates that the difference is statistically significant.

We will perform the following subgroup analyses: sex, men versus women; age, comparing outcomes for participants aged ≤30 versus >30; injury characteristic, non-sports versus sports injury; and grade 1 versus grade 2 sprain. To estimate significance of the subgroup treatment effect modification we use a Wald test of the treatment by subgroup interaction term from a logistic regression model.

To evaluate effects of missing data,we will employ appropriate data imputation methods to minimize bias. Specifically, we plan to use multiple imputation techniques, which allow us to create several complete datasets by filling in missing values based on observed data patterns. This approach will help us to better estimate the parameters of interest while accounting for the uncertainty associated with missing data.

### Ethics statement

The study protocol was approved by the Regional Ethics Review Committee of Shuguang Hospital (number: 2024-1535-118-01). All participants will provide their written consent. Trial registration: The trial was registered at Chinese Clinical Trial Registry https://www.chictr.org.cn/showproj.html?proj=234465 (Registration No.: ChiCTR2400087456). Date: 2024-07-29.

## Discussion

The TCAWM intervention for post-acute ankle sprains has the advantages of high acceptability and low pain, which can reduce the use of other drugs, thereby reducing risks and costs. The study aims to evaluate effectiveness and safety. Unfortunately, although there are many acupuncture studies on ankle sprains, however, studies on comprehensive treatment interventions in clinical practice have been rarely performed. To the best of our knowledge, this is the first trial exploring whether non pharmacological comprehensive intervention treatment that integrated traditional Chinese and western medicine rehabilitation programs will provide evidence on the optimal option of post-acute ankle sprain patients.

The purpose of this study is to investigate the impact of TCAWM intervention on the function of post-acute ankle sprains, and to clarify the specific role of the collaborative plan in the treatment of post-acute ankle sprains. The TCAWM intervention includes physical therapy, exercise training, acupuncture, and massage. Among them, physical therapy includes near-infrared and shock waves, the former can reshape the microenvironment and promote tissue regeneration [46], the latter promotes pro-inflammatory, catabolic and repair processes [47,48]; exercise training enhances the stability of the ankle joint and postural control through training in muscle strength, joint range of motion, proprioception, and balance function [49–52]; Acupuncture is used to relieve pain and inflammation. The analgesic effect of acupuncture is thought be due to the gate control theory and endogenous release of opioids [53]. Massage include pressing, kneading, stretching, shaking, rubbing, etc. Literature shows that massage helps overall sensorimotor function by stimulating sensory receptors, which is often quantified as postural control [52,54,55].

The evaluation method used in this study is currently recognized as the most suitable method. Meanwhile, this study has been registered at Chinese Clinical Trial Registry, which makes it more transparent and trustworthy. We hope to provide more treatment options for patients with post-acute ankle sprain, and encourage more experts and doctors in the same industry to carry out relevant research as much as possible. Our research also has limitations, and we hope to conduct more comprehensive, detailed, and in-depth research in future studies.

### Limitation

Despite the potential strengths, there are some limitations in our trial. Firstly, in this study, the intervention is a non-drug therapy, which cannot be ideally implemented in accordance with the provisions of double-blind. However, the strict management of researchers, data managers and statisticians in this study will help us to reduce performance bias. Secondly, the planned short treatment period to minimize dropout rates may affect the results of this study.Thirdly, due to insufficient preliminary data to determine the sample size, we have designed this experiment as a single center preliminary study. In addition, the sample selection for this protocol is between the ages of 18 and 35, further studies in various groups with a greater age range such as childern and olders are recommended. Fourthly, the patients cannot be blinded due to the particularity of treatment. Patients will know whether they had received additional treatment, which, in some cases, may have increase the risk of dropout in control patients. Finally, this is an exploratory study that does not involve long-term follow-up to observe long-term treatment outcomes and adverse reactions. For this reason,we will add a plan for participant follow-up after the protocol ends which could help assess the durability of intervention effects.

Nevertheless, the results of this study are expected to provide preliminary evidence on the effectiveness and acceptability of the integrated traditional Chinese and Western medicine in the treatment of grade I and grade II post-acute ankle sprains, and serve as the basis for further research.

The authors appreciate their colleagues in Shuguang Hospital Affiliated to Shanghai University of Traditional Chinese Medicine for their full support in recruiting and treating patients. The authors appreciate the efforts and help of investigators participating in this trial. The authors appreciate Jiming Tao for his guidance.

## Supporting information

S1 File Reporting checklist for protocol of a clinical trial. (DOCX)

S2 File Ethics statement document. (DOCX)

S3 File Ethical approval of clinical research plan. (DOCX)

S4 File Study protocol proofs. (DOCX)
