# Supplementary material for: The effects of integrated traditional Chinese and western medicine rehabilitation programs on post-acute ankle sprain: A randomized controlled trial study protocol
Source: PLoS One. 2025 Jan 30;20(1):e0318535. doi: 10.1371/journal.pone.0318535 (PMC11781713; doi:10.1371/journal.pone.0318535)
Supplement: S2 File — (DOCX) [file pone.0318535.s002.docx]

**中西医结合康复方案干预急性后踝关节扭伤的随机对照临床研究**

**1.研究目的**

客观评价中西医结合康复方案干预急性后踝关节扭伤临床有效性。

**2.研究对象**

**2.1一般资料**

选取上海中医药大学附属曙光医院康复医学科住院及门诊急性后踝关节扭伤患者。

**2.2样本量估算**

参考计量资料两样本均数比较样本含量估计公式，参考相关文献^[a]^试验组均值μ_2_=0.04，对照组μ_1_=0.25,σ=0.45，α=0.05，β=0.2，计算n=72，考虑20%脱落率，最终算的样本量为每组87例。


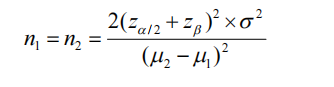


*a.张红阳. 新伤药外敷联合Mulligan技术对急性踝关节扭伤的疗效研究[D].西安体育学院,2023.000127.*

**2.3受试者须符合的标准**

**（1）诊断标准**

参照国家中医药管理局颁布的《中医病症诊断疗效标准》^[b]^及踝韧带扭伤美国物理治疗协会骨科分会功能、残疾和健康国际分类相关临床实践指南（2021版）^[c]^拟定：（1）有明确的踝部外伤史；（2）扭伤后踝关节有明显的压痛，或者有皮下瘀斑，伴随肿胀、疼痛，负重受限伴跛行；（3）关节活动受限；（4）Ottawa原则阴性, X线摄片检查未见骨折、脱位。

[b]国家中医药管理局.中医病症诊断疗效标准[M].南京:南京大学出版社,1994:64-65.

[c]Martin RL,Davenport TE,Fraser JJ,et al.Ankle Stability and Movement CoordinationImpairments:Lateral Ankle Ligament Sprains Revision 2021[J].J Orthop Sports Phys Ther.2021;51(4):CPG1-CPG80

**（2）扭伤分级标准**

1级：无功能丧失，韧带纤维拉长，关节无不稳，无韧带松弛（前抽屉试验和距骨倾斜测试阴性），很少或没有出血，无压痛点，踝关节的总活动度减少小于5°或更少，以及有踝关节肿胀小于0.5cm或更少。

2级：部分功能丧失，部分韧带纤维撕裂，关节轻度不稳，前抽屉试验阳性（涉及前距腓韧带），距骨倾斜测试阴性（没有涉及到跟腓韧带），出血，有压痛点，踝关节的总活动度减少大于5°，但小于10°，以及踝关节肿胀大于0.5cm但小于2cm。

3级：功能几乎完全丧失，韧带完全撕裂，关节明显不稳，前抽屉试验和倾斜测试阳性，皮下出血，压痛点非常明显，踝关节的总活动度下降大于10°，踝关节肿胀大于2cm。

**（3）纳入标准**

①符合上述诊断标准，首次运动扭伤两周后，且损伤分级为1、2级者；②年龄在18-35岁，性别不限；③坎伯兰踝关节不稳量表得分≤24分；④治疗前未经其他方法治疗且愿意接受保守治疗者，签署知情同意书。

**（4）排除标准**

①双踝均扭伤；②伤处局部有皮肤破损或者皮肤病者；③踝关节骨折、脱位、重度骨质疏松及可能影响肌肉力量的疾病，如糖尿病、类风湿性关节炎等；④患者主观不愿意接受试验者；⑤最近1周使用解热镇痛、安眠或激素类等药物；⑥有心脑血管、中枢神经肿瘤疾病、精神疾病史或头部外伤史；⑦踝部内有金属物等理疗禁忌者。

**（5）剔除标准**

①纳入后未接受后续评估与干预；②干预过程中接受额外治疗者；③各种原因引起其他系统疾病而不适宜继续治疗者；④发生其他情况，研究者认为不适宜继续参加本临床实验者。

**（6）脱落标准**

①未完成试验，未按时随访者；②未按给出方案实施诊治，或患者自行借助其他形式而不能界定成效者。

**3研究方法**

**3.1分组**

采用随机对照研究分为对照组和试验组，对照组采用常规治疗^[d]^，试验组采用曙光中西协同康复方案，每天1次，每周5次，共干预2周。

[d]Vuurberg G, Hoorntje A, Wink LM, et al. Diagnosis, treatment and prevention of ankle sprains: update of an evidence-based clinical guideline. Br J Sports Med. 2018;52(15):956. doi:10.1136/bjsports-2017-098106.

**3.2干预方案**

**3.2.1对照组**

常规治疗：包括加压冷敷、冲击波、微波等理疗；软组织牵伸放松、踝关节被动及主动关节活动、踝关节周围肌肉力量训练。

**3.2.2试验组**

曙光中西协同康复方案:①理疗：红外线剂量300w，距离20cm，踝部照射；冲击波能流密度0.15mJ/cm2，脉冲数2000次。②运动训练：a.肌力训练：利用弹力带进行踝背伸、跖屈、内翻和外翻抗阻训练，保持10秒，各个方向进行20次，每天2组；b.关节活动度训练：受试者进行踝关节主动背伸、跖屈、内翻及外翻，运动到踝关节最大范围时保持10秒，各个方向各运动20次；并进行踝关节顺时针及逆时针旋转运动，动作柔和、缓慢、速度均匀，各旋转20圈。每天2组；c.平衡训练板训练：站立在平衡板上，双足无额外支撑下保持30s，训练20次再进行单足无额外支撑下保持平衡20s，双足交替进行，各训练20次，每天2组；d.稳定强化训练：进行原地起跳训练，折返跑（距离100m），每组起跳训练50次，折返跑10次（一来一回计1次）。每天2组。③针灸：以局部穴位为主穴，配穴选择足少阳经筋及阳跷脉病证配丘墟、足临泣、申脉；足太阴经筋及阴跷脉病证配商丘、照海、水泉。毫针刺用泻法，或在肿胀局部阿是穴行围刺法；可用温针灸、电针。④推拿：选择踝关节周围及阳陵泉、丘墟、绝骨、照海、申脉等穴位。手法采用按、揉、一指禅推、拔伸、摇、擦等。患者取仰卧位，医师站于患侧，以拇指按揉法作用于踝部，先从患部到周围，再从外踝经小腿外侧至阳陵泉穴，按揉3遍，重点在阳陵泉、丘墟、绝骨、照海、申脉等穴位，以酸胀为度；后以一指禅推法作用于痛处，从局部向周围扩散。最后拔伸踝关节数次，并作小幅度内外旋动；继而做踝关节摇法数次；以小鱼际擦法擦足背部，并经踝至小腿，以温热为度。

**3.3观察指标**

（1）主要结局指标

①视觉模拟评分法（VAS）。

VAS是用来评估疼痛程度。在纸上划一条起端为0终端为10的10cm的橫线，标记0为无痛，10为不能忍受的疼痛，横线中间每1cm都制定好相应刻度的标记。让受试者根据疼痛感觉在橫线上任意位置做标记，作为受试者VAS疼痛的程度。

②简明McGill疼痛问卷（SF-MPQ）。

SF-MPQ用来评估疼痛感觉，共有47项，前11项评估疼痛感觉程度（PRIA），12~15项评估对疼痛情感状况（PRIB）。每项的疼痛程度描述为无痛（0分），轻度（1分），中度（2分），重度（3分）。此外，同时现在疼痛状况和视觉模拟评分纳入对总体疼痛状况的评估。

（2）次要结局指标

①足踝能力评估量表（FAAM）。

FAAM是评定日常生活能力限制程度和足踝活动障碍程度的方法，由日常生活活动的21项得分和独立运动障碍量表的8项得分组成。

②AOFAS踝-后足量表。

采用美国骨科足踝协会(AOFAS)踝-后足量表，作为检查踝关节韧带损伤的一个可靠有效的工具。该标准由两大部分组成，主要包括疼痛、功能和自主活动、支撑情况、屈/伸、内/外翻活动、踝足稳定性等，满分100分。两大部分评分各为50分，满分100分，优(90~100分)，良(75~89分)，一般(50~74分)，差(<50分)，分值越大表明足踝关节功能、症状越好。

③力矩：峰力矩（PT）；峰力矩体重比（PT/BW）；平均功率（AP）；屈伸峰力矩比值（F/E）；同名异侧肌比值。

测试仪器选用美国BIODEX多关节等速力量测试和训练系统，测试得出的原始数据由计算机自动生成。受试者在座椅上保持仰卧位，根据其身高、体型等，严格按照设备安全使用手册调整并固定设备。测试时，按照软件所提供的参数进行座椅高度和动力头刻度调节，将足移动平面和足部踏板移动平面重合，外踝与动力头转动中心保持在一条直线上，使用尼龙绳将受试者大腿和足固定在配件上，测试前进行重力补偿。首先，在角速度为60°/s条件下，让受试者进行踝关节背伸及跖屈3次最大收缩练习，当受试者熟悉后，休息30min依次进行健、患侧踝关节等速肌力测试，分别在角速度为30°/s、60°/s、120°/s条件下，进行踝关节背伸及跖屈、踝关节内翻及外翻的主动向心运动，每次完成10次连续重复的最大收缩。

④步态：步长；步速；步频；支撑相；足底压力；踝屈曲、内翻角度。

应用脉沃Odonate三维运动捕捉与步态分析设备测试患者的步行能力，足底压力。采集端选择步态采集结合足底压力检测模式。基于国际生物力学学会所推荐的工作框架和贴点模型。受试者需穿紧身衣裤。红外线反光点（Marker）定位（共23个）。全部过程均由同一位资深康复治疗师完成操作。采集前指示患者行走区域，患者行走区域为距离摄像臂1.6米至4.5米处。平置摄像臂，并确定摄像臂平行于地面后。首先指示患者在2米左右位置处静止站立，点击“静态采集”；然后指示患者在4.5米左右位置处站立，点击“动态采集”按钮，指示患者在原地挥动手臂后开始正常在步道中行走。

**3.4数据管理与监控**

病例报告表（CRF）包括观察时间点，结局指标，不良事件和安全性评估。医生处于盲态下进行各种临床疼痛指标的评估，按照CRF的要求及时准确地填写相关信息。只有结果评估者才能访问CRF并执行数据输入。

**3.5统计分析**

使用SPSS21.0统计软件进行统计分析，描述性统计：包括均数、标准差等。计量资料采用方差分析等方法。计数资料采用卡方检验、Fisher精确检验等；等级资料采用非参数秩和检验、CMH卡方检验等。设α=0.05为显著性检验水准，当P<0.05时表明差异具有统计学意义。

**3.6技术路线**

**临床行为学统计分析**

中西医结合康复方案干预急性后踝关节扭伤对疼痛、运动能力等维度的改善作用

疼痛指标评估

VAS评分

McGill量表评分

踝运动功能指标评估

FAAM足踝能力量表

AOFAS踝-后足量表

生物力学指标评估

力矩、步态

中西医结合康复方案干预急性后踝关节扭伤的临床行为学效应研究

对照组87例

试验组87例

运动员踝扭伤患者174例

随机对照

纳入、排除标准

干预前后指标检测
