# Supplementary material for: The effects of integrated traditional Chinese and western medicine rehabilitation programs on post-acute ankle sprain: A randomized controlled trial study protocol
Source: PLoS One. 2025 Jan 30;20(1):e0318535. doi: 10.1371/journal.pone.0318535 (PMC11781713; doi:10.1371/journal.pone.0318535)
Supplement: S3 File — (DOCX) [file pone.0318535.s003.docx]

**A randomized controlled clinical study on the intervention of post-acute ankle sprain with integrated of traditional Chinese and Western medicine rehabilitation programs**

**1.** **Research objective**

Objective evaluation of the clinical effectiveness of the integrated traditional Chinese and Western medicine rehabilitation programs in intervening in post-acute ankle sprain.

**2.** **Research object**

**2.1** **General information**

Select inpatients and outpatient patients with post-acute ankle sprains from the Rehabilitation Medicine Department of Shuguang Hospital Affiliated to Shanghai University of Traditional Chinese Medicine.

**2.2** **Sample size estimation**

The formula for estimating sample size by comparing the mean values of two samples with reference to bibliometric data, and referring to relevant literature, the mean values of the experimental group μ_2_=0.04, and the control group μ_1_=0.25, σ=0.45，α=0.05，β=0.2， Calculate n=72, considering a 20% dropout rate, and the final sample size is 87 cases per group.

n_1_=n_2_=$2\times\frac{\left( Z_{\frac{\alpha}{2}}＋Z_{\beta} \right)^{2}\times{}^{2}}{（{\mu2-\mu1）}^{2}}$

**2.3** **Standards that subjects must meet**

**2.3.1 Diagnostic criteria**

Referring to the "Diagnosis and Efficacy Standards for Traditional Chinese Medicine Diseases" issued by the National Administration of Traditional Chinese Medicine and the Clinical Practice Guidelines for the International Classification of Function, Disability, and Health of the Orthopedic Branch of the American Physical Therapy Association for Ankle Ligament Sprains (2021 Edition), it is proposed that: (1) there is a clear history of ankle injury; (2) After a sprain, there is obvious tenderness or subcutaneous bruising in the ankle joint, accompanied by swelling and pain, limited weight-bearing, and limping; (3) Limited joint movement; (4) Ottawa principle is negative, and X-ray examination shows no fractures or dislocations.

**2.3.2 Ankle sprain grading standards**

Grade 1: No loss of function, elongation of ligament fibers, no instability of joints, no ligament relaxation (negative for anterior drawer test and talus tilt test), little or no bleeding, no tenderness points, decreased total ankle range of motion less than 5 ° or less, and ankle swelling less than 0.5cm or less.

Grade 2：Partial loss of function, partial ligament fiber tearing, mild joint instability, positive anterior drawer test (involving anterior talofibular ligament), negative talus tilt test (not involving calcaneofibular ligament), bleeding, tenderness points, decreased total ankle range of motion greater than 5 ° but less than 10 °, and ankle swelling greater than 0.5cm but less than 2cm.

Grade 3：Almost complete loss of function, complete tearing of ligaments, significant joint instability, positive anterior drawer test and tilt test, subcutaneous bleeding, significant tenderness points, total range of motion of ankle joint decreased by more than 10 °, ankle joint swelling greater than 2cm.

**2.3.3 Inclusion criteria**

① Those who meet the above diagnostic criteria, have suffered a sprain two weeks after their first exercise, and have been graded as level 1 or level 2; ② Age range from 18 to 35 years old, regardless of gender; ③ Cumberland Ankle Instability Scale score ≤ 24 points; ④ Those who have not received any other treatment before treatment and are willing to receive conservative treatment shall sign an informed consent form.

**2.3.4 Exclusion criteria**

① Both ankles sprained; ② Individuals with skin damage or skin disease in the affected area; ③ Ankle fracture, dislocation, severe osteoporosis and diseases that may affect muscle strength, such as diabetes and rheumatoid arthritis; ④ Subjectively unwilling to accept the experimenter by the patient; ⑤ Use antipyretic, analgesic, sedative, or steroid drugs in the past week; ⑥ Have a history of cardiovascular and cerebrovascular diseases, central nervous system tumor diseases, mental illness, or head injury; ⑦ Individuals with contraindications for physical therapy such as metal objects in the ankle.

**2.3.5 Dropout criteria**

① Those who have not completed the experiment and have not been followed up on time; ② Failure to implement diagnosis and treatment according to the given plan, or the patient's inability to define the effectiveness through other means on their own.

**3** **Research method**

**3.1 Grouping**

A randomized controlled study was conducted and divided into a control group and an experimental group. The control group received routine treatment, while the experimental group received Shuguang Chinese Western collaborative rehabilitation programS, once a day and five times a week, for a total of two weeks of intervention.

**3.2 Intervention**

**3.2.1 The control group**

Routine intervention: including pressure cold compress, shock wave, microwave and other physical therapies; Soft tissue stretching and relaxation, passive and active joint activity of the ankle, and strength training of muscles around the ankle.

**3.2.2 The experimental group**

TCAWM intervention：

1. Physical therapy: infrared dose of 300w, distance of 20cm, irradiate the sick ankle; The shockwave, energy flow density is 0.15mJ/cm2, with a pulse count of 2000.
2. Exercise training: a. Muscle strength training: Use elastic bands for ankle dorsiflexion, plantar flexion, inversion, and eversion resistance training, maintain for 10 seconds, perform 20 times in all directions, 2 groups per day; b. Joint range of motion training: Participants engage in active ankle dorsiflexion, plantar flexion, inversion, and eversion, maintaining a maximum range of motion for 10 seconds, and performing 20 movements in each direction; And perform clockwise and counterclockwise rotation movements of the ankle joint, with gentle, slow, and uniform speed, each rotating 20 times. Two groups per day; c. Balance training board training: Stand on the balance board, maintain balance with both feet without additional support for 30 seconds, train 20 times, and then maintain balance with one foot without additional support for 20 seconds, alternating between the two feet, each training 20 times, with 2 groups per day; d. Stable reinforcement training: Conduct in place takeoff training, turn back runs (distance of 100m), each group of takeoff training 50 times, turn back runs 10 times (once in a row). Two groups per day.
3. Acupuncture: local acupoints are the main acupoints, and the acupoints selected are GB40, GB41, BL62, SP5, KI6 and KI5. Warm acupuncture and moxibustion and electroacupuncture can be used.
4. Massage: Select acupoints around the ankle joint, such as GB34, GB40, GB39, KI6, and BL62. The techniques used include pressing, kneading, one finger Zen pushing, stretching, shaking, rubbing, etc. The patient is placed in a supine position, with the physician standing on the affected side and using the thumb massage method to apply pressure to the ankle. First, from the affected area to the surrounding area, and then from the outer ankle through the outer side of the calf to GB34 acupoint, massage three times, with a focus on GB34, GB40, GB39, KI6, BL62 and other acupoints, with a degree of soreness and swelling. Finally, pull and extend the ankle joint several times and perform small internal and external rotations; Then perform ankle joint rocking several times; Wipe the back of the foot using the small thenar wiping method, and extend from the ankle to the calf.

**3.3 Outcome measures**

**3.3.1Primary outcomes**

1. Visual Analog Scale (VAS). VAS is used to evaluate the degree of pain. Draw a 10cm horizontal line on the paper with a starting point of 0 and a ending point of 10, marking 0 as painless and 10 as unbearable pain. Mark the corresponding scale every 1cm in the middle of the line. Ask the subjects to mark any position on the horizontal line based on their pain perception, as the degree of VAS pain in the subjects.
2. Short form McGill Pain Questionnaire (SF-MPQ). SF-MPQ is used to evaluate pain sensation, with a total of 47 items. The first 11 items evaluate the degree of pain sensation (PRIA), and the 12-15 items evaluate the emotional state of pain (PRIB). The pain level of each item is described as painless (0 points), mild (1 point), moderate (2 points), and severe (3 points). In addition, both pain status and visual analogue scores are now included in the evaluation of overall pain status.

**3.3.2 Secondary indicators**

1. Foot and Ankle Ability Assessment Scale (FAAM). FAAM is a method for assessing the degree of daily living ability limitation and ankle mobility disorders, consisting of 21 scores from daily living activities and 8 scores from the Independent Movement Disorder Scale.
2. AOFAS Ankle Posterior Foot Scale. Using the American Orthopedic Foot and Ankle Association (AOFAS) Ankle Posterior Foot Scale as a reliable and effective tool for detecting ankle ligament injuries. This standard consists of two main parts, mainly including pain, function and autonomous activity, support, flexion/extension, inward/outward rotation, ankle and foot stability, etc., with a maximum score of 100 points. The two major scores are each 50 points, with a maximum score of 100 points, excellent (90-100 points), good (75-89 points), average (50-74 points), and poor (<50 points). The larger the score, the better the ankle joint function and symptoms.
3. Torque: Peak torque (PT); Peak torque to body weight ratio (PT/BW); Average power (AP); The ratio of bending to elongation peak torque (F/E); The ratio of homonymous contralateral muscles. The testing instrument uses the BIODEX multi joint constant velocity force testing and training system from the United States, and the raw data obtained from the test is automatically generated by the computer. The subject is in a supine position on the seat, and according to their height, body shape, etc., the equipment is adjusted and fixed strictly in accordance with the equipment safety manual. During testing, adjust the seat height and power head scale according to the parameters provided by the software, align the foot movement plane with the foot pedal movement plane, keep the outer ankle in a straight line with the power head rotation center, use nylon ropes to fix the subject's thighs and feet to the accessories, and perform gravity compensation before testing. Firstly, under the condition of an angular velocity of 60 °/s, the subjects were subjected to three maximum contraction exercises of ankle dorsiflexion and plantarflexion. After familiarizing themselves, the subjects rested for 30 minutes to sequentially perform isokinetic muscle strength tests on the healthy and affected ankle joints. Under the conditions of an angular velocity of 30 °/s, 60 °/s, and 120 °/s, active centripetal movements of ankle dorsiflexion and plantarflexion, ankle inversion, and eversion were performed, with 10 consecutive maximum contractions completed each time.
4. Gait: Step length; Step speed; Step frequency; Supporting phase; Plantar pressure; Ankle flexion and inversion angle. Apply Maiwo Odonate 3D motion capture and gait analysis equipment to test the patient's walking ability and plantar pressure. The collection end selects gait collection combined with plantar pressure detection mode. Based on the work framework and patch model recommended by the International Society of Biomechanics. The subjects are required to wear tight fitting clothing. Infrared reflector positioning (23 in total). The entire process is carried out by the same senior rehabilitation therapist. Before collection, indicate the patient's walking area, which is 1.6 to 4.5 meters away from the camera arm. Place the camera arm horizontally and ensure that it is parallel to the ground. Firstly, instruct the patient to stand still at a position of about 2 meters and click on "Static Collection"; Then instruct the patient to stand at a position of about 4.5 meters, click the "Dynamic Collection" button, and instruct the patient to start walking normally on the trail after waving their arms in place.

**3.4 Data management and monitoring**

The Case Report Form (CRF) includes observation time points, outcome indicators, adverse events, and safety assessments. Fill in relevant information promptly and accurately according to the requirements of CRF. Only result evaluators can access CRF and perform data input.

**3.5 Statistical analysis**

Use SPSS 21.0 statistical software for statistical analysis, including descriptive statistics such as mean, standard deviation. Quantitative data are analyzed using methods such as analysis of variance. Count data were analyzed using chi square test, Fisher's exact test; Non parametric rank sum test and CMH chi square test were used for rank data. Set α=0.05 as the significance test level, and when P<0.05, it indicates that the difference is statistically significant.
