# Supplementary material for: The effects of integrated traditional Chinese and western medicine rehabilitation programs on post-acute ankle sprain: A randomized controlled trial study protocol
Source: PLoS One. 2025 Jan 30;20(1):e0318535. doi: 10.1371/journal.pone.0318535 (PMC11781713; doi:10.1371/journal.pone.0318535)
Supplement: S4 File — (DOCX) [file pone.0318535.s004.DOCX]

Notice on the Announcement of the List of Key Supporting Discipline Construction Projects in Shanghai's Health System for the Year 2023

Related units:

In order to further improve the disciplinary system, strengthen support for disciplines such as general medicine, cultivate competitive disciplinary advantages, and cultivate scarce professional talents from point to area, the Municipal Health Commission has launched the application work for key supported disciplines in 2023. After expert review and comprehensive evaluation, as well as consultation with the Municipal Financial Science and Technology Investment Coordination Platform, we hereby announce the funding projects for key supported discipline construction projects in 2023 (see attachment). Each project will receive a financial subsidy of 1 million yuan, and the final funding will be subject to budget review. The project undertaking unit shall provide no less than 30% of the supporting facilities. The project construction period is from September 2023 to August 2026.

Please attach great importance to this work in all project units and higher-level supervisory departments, actively implement the construction project, focus on strengthening goal management and process management, and ensure the implementation of the established goals and priorities.

This is to inform you.

Attachment: List of Key Supported Discipline Construction Projects for the Year 2023

Shanghai Municipal Health Commission

September 15, 2023

Attachment

List of Key Supported Discipline Construction Projects for 2023

| No. | Discipline | Undertaking unit | Person in charge | Funding support (10000 yuan) |
| --- | --- | --- | --- | --- |
| 12 | Rehabilitation | Shuguang Hospital Affiliated to Shanghai University of Traditional Chinese Medicine | Jiming Tao | 100 |

information sources:

<https://wsjkw.sh.gov.cn/kjjy2/20230918/4cb8dcad0faf4be78d88e3ebe95d28c6.html>

STCSM2023-12-06 16：39：11

**Technology Plan Project Contract**

**Project number：23DZ1204000**

**Project name：Research on the Diagnosis, Treatment, and Evaluation of Common Sports Injuries through the Integration of Traditional Chinese and Western Medicine**

**Project Undertaking Unit: Shanghai University of Traditional Chinese Medicine**

**Project leader：Xinlin Chen**

**Project execution period：2024.01.01-2026.12.31**

**Science and Technology Commission of Shanghai Municipality**

STCSM2023-12-06 16：39：11

**Client（Party A）：Science and Technology Commission of Shanghai Municipality**

**Joint funding units(Party B):** **Shanghai Municipal Sports Bureau**

**Undertaking unit (Party C):** **Shanghai University of Traditional Chinese Medicine**

The three parties, A, B, and C, sign this contract in accordance with the "Shanghai Science and Technology Plan Project Management Measures", "Shanghai Science and Technology Plan Project (Project) Special Fund Management Shanghai Science and Technology Plan Project Comprehensive Performance Evaluation Work Standards (Trial)" and other documents, as well as relevant laws and policies. We agree to exercise the rights and obligations stipulated in this contract with good faith, and the relevant content serves as an important basis for project implementation management, supervision, and evaluation.

**1、 Basic information of the project:**

The three parties to the contract unanimously agree to the content of the project task book attached to this contract, and strictly implement the project in accordance with the research content and assessment indicators of the project task book.

Project number：23DZ1204000

Project name：Research on the Diagnosis, Treatment, and Evaluation of Common Sports Injuries through the Integration of Traditional Chinese and Western Medicine

Project Undertaking Unit: Shanghai University of Traditional Chinese Medicine

Project leader：Xinlin Chen

Project execution period：2024.01.01-2026.12.31

(3) Project 3:

Number：23DZ1204003

Name：A randomized controlled clinical study on the intervention of integrated traditional Chinese and Western medicine rehabilitation program for ankle sprains in athletes

Undertaking Unit: Shuguang Hospital Affiliated to Shanghai University of Traditional Chinese Medicine

Leader：Weian Yuan


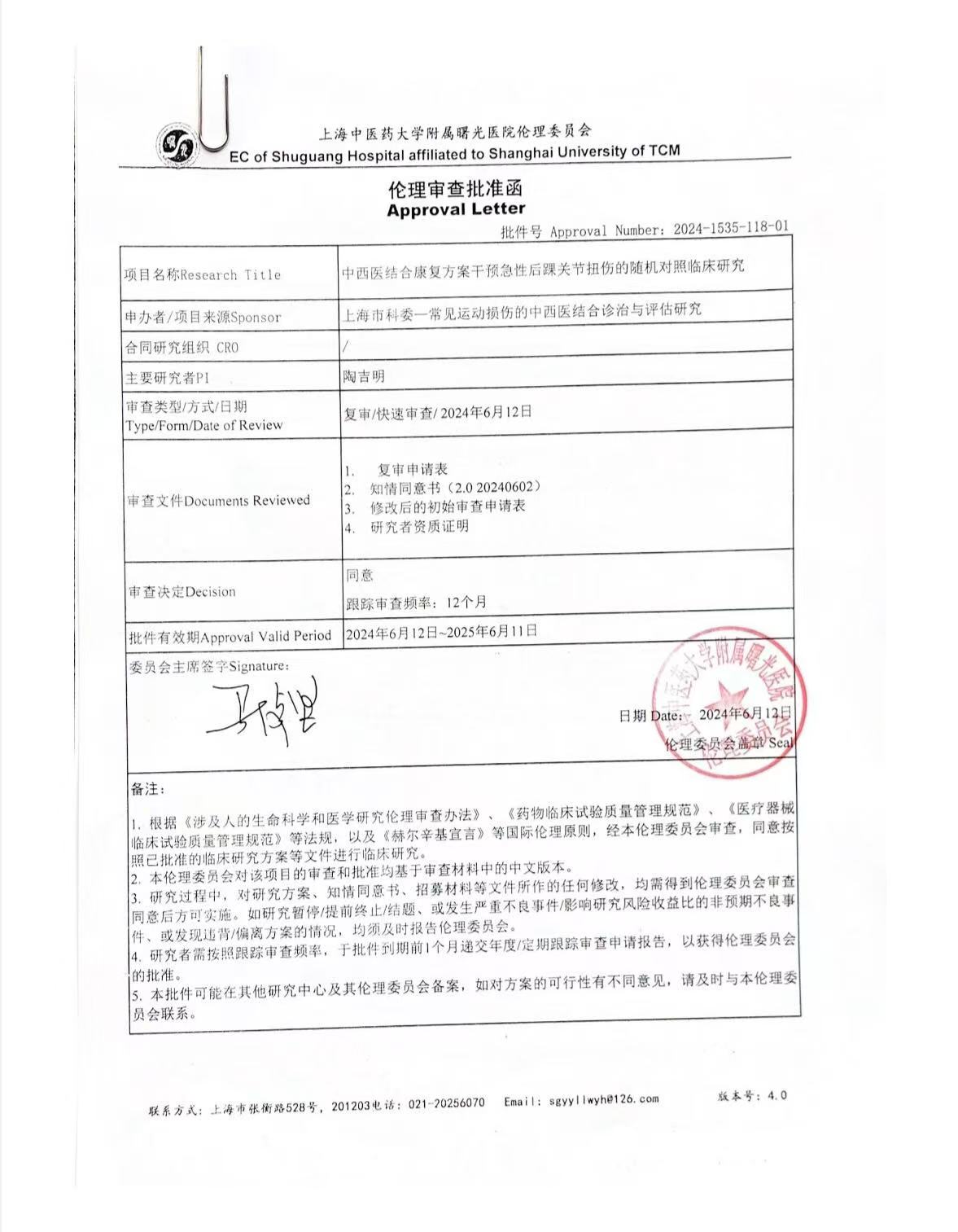


**Approval Letter**

Approval Number：2024-1535-118-01

| Research Title | A randomized controlled clinical study on the intervention of post-acute ankle sprain with integrated of traditional Chinese and Western medicine rehabilitation programs |
| --- | --- |
| Sponsor | Shanghai Municipal Commission of Science and Technology - A Study on the Diagnosis, Treatment and Evaluation of Common Sports Injuries by Combining Traditional Chinese and Western Medicine |
| Contract research organization（CRO） | / |
| Principal investigator（PI） | Jiming Tao |
| Type/Form/Date of Review | Review/Rapid Review/June 12, 2024 |
| Documents Reviewed | 1. Reexamination application form  2. Informed consent form (2.0, 20240602)  3. Revised initial review application form  4. Qualification Certificate of Researchers |
| Decision | Agree  Tracking review frequency: 12 months |
| Approval Valid Period | June 12, 2024 to June 11, 2025 |
| Remarks:  1. According to regulations such as the "Ethical Review Measures for Human Life Sciences and Medical Research", "Good Clinical Practice for Drug Trials", "Good Clinical Practice for Medical Devices", and international ethical principles such as the "Helsinki Declaration", after review by this ethics committee, it is agreed to conduct clinical research in accordance with the approved clinical research protocol and other documents.  2. The review and approval of this project by this ethics committee are based on the Chinese version of the review materials.  During the research process, any modifications made to the research protocol, informed consent form, recruitment materials, etc. must be reviewed and approved by the ethics committee before implementation. If the research is suspended/terminated early/concluded, or serious adverse events/unexpected adverse events that affect the risk return ratio of the research occur, or any deviations/deviations from the protocol are found, they must be reported to the ethics committee in a timely manner.  4. Researchers are required to submit annual/periodic follow-up review application reports one month before the approval deadline in accordance with the frequency of follow-up review, in order to obtain approval from the ethics committee.  5. This approval may be filed with other research centers and their ethics committees. If you have different opinions on the feasibility of the plan, please contact our ethics committee in a timely manner. | |

Contact information: No. 528 Zhangheng Road, Shanghai，201203 Tel：021-20256070 Email：[sgyyllwyh@126.com](mailto:sgyyllwyh@126.com) Version number:4.0

EC of Shuguang Hospital affiliated to Shanghai University of TCM
